# Comparative Study of the Electrochemical, Biomedical, and Thermal Properties of Natural and Synthetic Nanomaterials

**DOI:** 10.1186/s11671-018-2508-3

**Published:** 2018-04-20

**Authors:** Ferial Ghaemi, Luqman Chuah Abdullah, Hanieh Kargarzadeh, Mahnaz M. Abdi, Nur Farhana Waheeda Mohd Azli, Maryam Abbasian

**Affiliations:** 1 Institute of Tropical Forestry and Forest Products (INTROP), Universiti Putra Malaysia (UPM), 43400UPM Serdang, Selangor Malaysia; 2 Department of Chemical and Environmental Engineering, Universiti Putra Malaysia (UPM), 43400UPM Serdang, Selangor Malaysia; 3 Faculty of Science and Technology, School of Chemical Sciences and Food Technology, Polymer Research Center (PORCE), Universiti Kebangsan Malaysia (UKM), 43600 Bangi, Selangor Malaysia; 4 Department of Chemical Engineering, Shiraz University, Shiraz, 7134851154 Iran; 5 Department of Chemistry, Faculty of Sciences, Universiti Putra Malaysia (UPM), 43400UPM Serdang, Selangor Malaysia; 6 Medical Toxicology Research Center, School of Medicine, Mashhad University of Medical Science, Mashhad, Iran

**Keywords:** Natural and synthetic nanomaterials, Electrochemical properties, Cytotoxicity effect, Thermal stability

## Abstract

In this research, natural nanomaterials including cellulose nanocrystal (CNC), nanofiber cellulose (NFC), and synthetic nanoparticles such as carbon nanofiber (CNF) and carbon nanotube (CNT) with different structures, sizes, and surface areas were produced and analyzed. The most significant contribution of this study is to evaluate and compare these nanomaterials based on the effects of their structures and morphologies on their electrochemical, biomedical, and thermal properties. Based on the obtained results, the natural nanomaterials with low dimension and surface area have zero cytotoxicity effects on the living cells at 12.5 and 3.125 μg/ml concentrations of NFC and CNC, respectively. Meanwhile, synthetic nanomaterials with the high surface area around 15.3–21.1 m^2^/g and significant thermal stability (480 °C–600 °C) enhance the output of electrode by creating a higher surface area and decreasing the current flow resistance.

## Background

Natural nanomaterials involve nanocellulose in different shapes such as cellulose nanofiber (NFC) and cellulose nanocrystal (CNC). Roughly, individual cellulose molecular chains connect to each other through hydrogen bonds to form bigger units known as rudimentary fibrils or microfibrils [1]. These microfibrils have some amorphous areas and exceedingly ordered (crystalline) areas. When microfibrils are divided into nanometer particles, the nanofibrils are formed. The nanofibrils domains generally referred to as nanocellulose, are a promising raw material for new bio-based composites because of their high mechanical strength, stiffness, low thermal expansion, large surface area, renewability, optical transparency, biodegradability, and low toxicity [2].

There are many natural sources used to prepare nanocellulose. Kenaf is a natural tropical plant that has been grown commercially to generate a secondary source of income for developing countries including Malaysia [3]. The high cellulose content ranging between 44 and 63.5% in kenaf has generated interest for many applications [3, 4]. The CNC and NFC can be obtained via acid hydrolysis and mechanical treatment, respectively. Because of their excellent characteristics such as nontoxicity, high electrical, and also thermal properties, they have been used in many fields such as filler in polymer composite to create an extensive variety of other functional materials, such as transparent barrier films [5], photonic crystals [6], shape-memory polymers [7], drug carriers [8], and composite materials [9].

Synthetic nanomaterials including carbon nanomaterials have many applications in industries and sciences [10–12]. Carbon nanomaterials such as carbon nanotube (CNT) and carbon nanofiber (CNF) are made from sp^2^ carbon atoms with one-dimension (1D) structures [10]. The structure of pure CNT can be visualized as a single sheet of graphite rolled to form a tube. The properties of nanotubes depend on the atomic arrangement, the diameter and length of the tubes, and the morphology or structure [13]. Besides, CNFs have cylindrical nanostructures with different stacking arrangements of graphene sheets such as stacked platelet, ribbon, or herringbone [11, 14]. They have diameter varies between some tens of nanometers up to several hundreds of nanometers, while their lengths are in the order of micrometers [14]. Carbon nanomaterials with low-density and high-aspect ratio, as well as extraordinary mechanical, thermal, electrical, and electrochemical properties, have been used in many activities in most areas of science and engineering [15]. Besides, in many cases, these nanomaterials have many applications in biomedical fields [12, 16, 17]. Although there are several techniques to produce CNT and CNF including arc-discharge [18], laser ablation [19], chemical vapor deposition (CVD) [20–23], and self-assembly [24]. CVD as the large-scale production method has been used to produce high-quality CNT and CNF [25]. In order to obtain different morphology, some important parameters of CVD such as runtime, reaction temperature, carbon source flow rate, and catalyst concentration should be altered [26–29].

To the best of the researchers’ knowledge, so far, nobody has reported any research on a comparison study of the properties of natural and synthetic nanomaterials. Here, the main objective is to compare different forms of nanocellulose and nanocarbon in terms of their structure, morphology, composition, crystallization, surface area, and also thermal stability, cytotoxicity effects, and electrochemical properties. The Brunauer, Emmet, and Teller analysis (BET) was applied to measure the specific surface area. The surface morphology, composition, and structural characterization of the samples were analyzed through scanning electron microscopy (SEM), energy dispersive X-ray (EDX), transmission electron microscope (TEM), and X-ray diffraction (XRD). Moreover, different analyses such as thermogravimetric analysis (TGA), cyclic voltammograms (CV), and MTT assay were applied to investigate the influences of the structure, composition, and morphology of the nanoparticles on their thermal, electrochemical, and toxicity properties.

## Results and Discussion

### Morphology of Nanomaterials

**Fig. 1:**
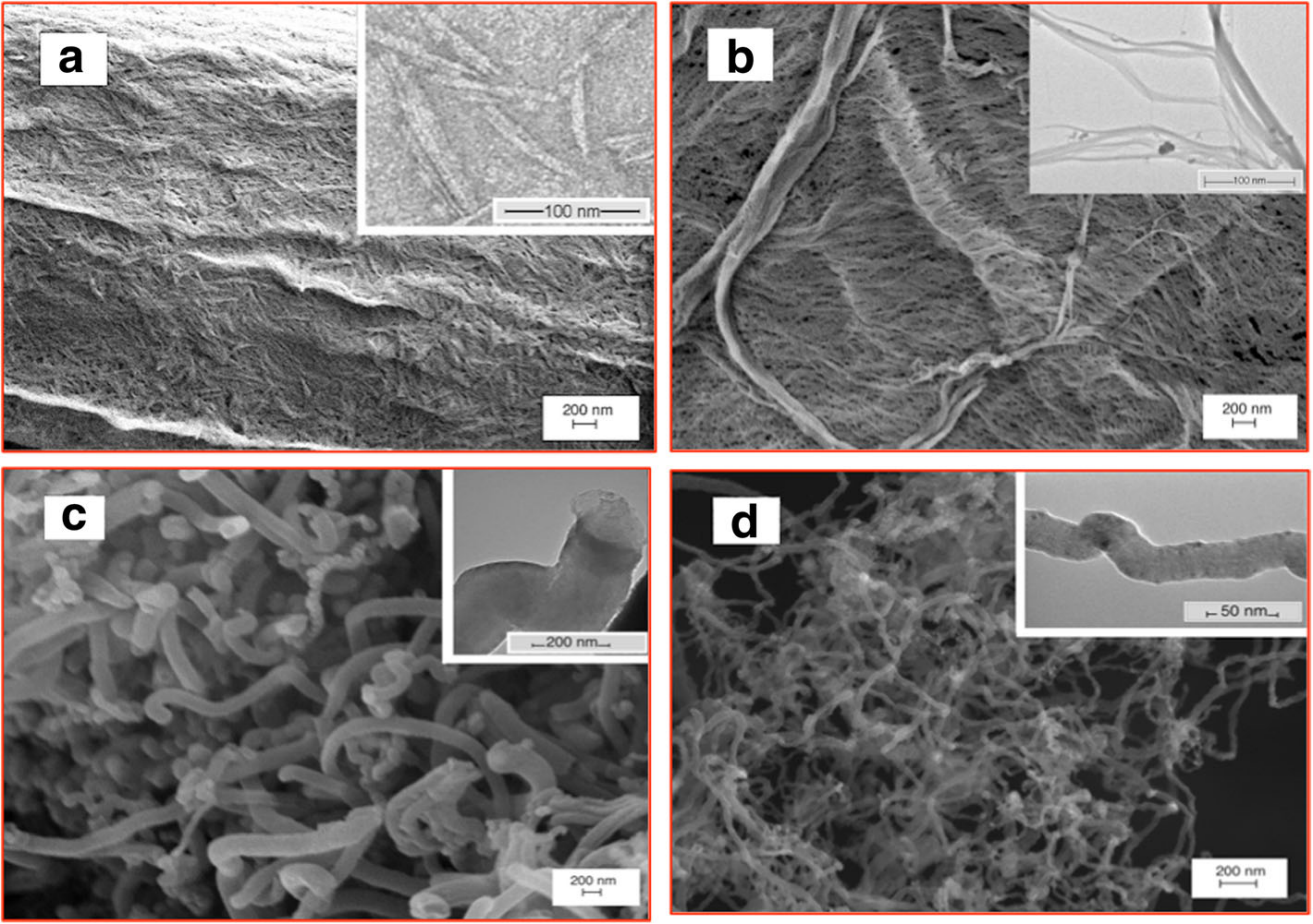
SEM/TEM images of **a** CNC, **b** NFC, **c** CNF, and **d** CNT

The SEM and TEM images in Fig. 1 depict the micrographs of the natural and synthetic nanomaterials. The nanomaterials show substantially different shapes and sizes in micrographs. To capture the TEM images of the nanoparticles, the sample was dispersed in an acetone solution in order to separate the nanoparticles from each other.

According to the images, CNCs present a needle-like structure with an average length of 150 nm and a diameter of 12 nm, whereas, NFCs exhibit a highly entangled, web-like structure with diameters ranging from 50 to 200 nm (see Fig. 1a,b). The highly entangled structure of NFCs significantly increased the resistance to flow and resulted in the gel-like behavior of the as-received NFC sample. Figure 1c reveals the CNF with rod-shaped structure and 150–200 nm in diameter has very coarse and solid surface while the CNTs on the surface are multi-walled, curly, and entangled with each other. Figure 1d shows that the wall thickness diameters of CNTs were about 10–30 nm. Similar to the NFCs, the lengths of CNT and CNF were too long and it was not easy to measure the length of individual fiber with high accuracy due to their entangle structures.

### Energy Dispersive X-ray Spectroscopy

In order to find the composition of each nanostructure, the EDX was employed. The EDX results of each type of nanomaterials are reported in Table 1. All nanoparticles revealed the presence of carbon and oxygen in huge amount. EDX result for CNC showed that not only carbon and oxygen but also the presence of a little amount of S, while the presence of contaminant for NFC was not reported, which was related to the preparation method. The EDX results of CNF and CNT synthesized with CVD method proved the presence of low amount of Ni catalyst in the products. Although the CNF and CNT were immersed in the FeCl_3_/HCl solution to remove Ni catalyst, a small amount of Ni related to the presence of Ni catalyst in the nanofiber and nanotubes was still observed. As expected, the produced nanomaterials possessed mostly carbon and oxygen and the number of impurities was low as reported in Table 1.

**Table 1:** EDX results for different nanomaterials

Sample	C (wt%)	O (wt%)	Others (wt%)
CNC	45.34	51.96	S (2.7)
NFC	47.95	52.05	0
CNF	56.41	43.09	Ni (0.5)
CNT	58.69	41.01	Ni (0.3)

### BET Surface Area

To acquire the surface activity of the natural and synthetic nanomaterials, the BET-specific surface area was determined. Table 2 reported the BET results obtained from the nitrogen adsorption-desorption isotherms. The nanomaterials were dried at 200 °C to remove humidity. Adsorption and desorption hysteresis indicates some pores existing in the nanostructures. According to the results, the BET surface areas for natural nanomaterials are lower than synthetic nanomaterials, which were proven by pore size and volume of the nanomaterials. The pore size and volume of the natural nanomaterials are much lower than pore size and volume of synthetics ones.

**Table 2:** BET results of natural and synthetics nanomaterials

Sample	BET surface area (m^2^/g)	Pore size (nm)	Pore volume (m^3^/g)
CNC	13.398	151.390	0.652314
NFC	9.756	173.524	0.792103
CNF	15.331	120.438	0.584305
CNT	21.154	54.965	0.347281

Therefore, the surface area of the nanocarbon was higher than the nanocellulose, which was due to the formation of carbon nanomaterials with not only small dimensions but also network structures. Besides, the difference in the morphology and diameter between the CNF and CNT led to the differential surface activity. Finally, it was found that among these nanoparticles, NFC had the lowest surface area, while CNT had the highest. Hence, the resultant CNT with the highest surface area had the potential to be used as pioneer nanoparticles for many applications such as absorptive composites.

### XRD

**Fig. 2:**
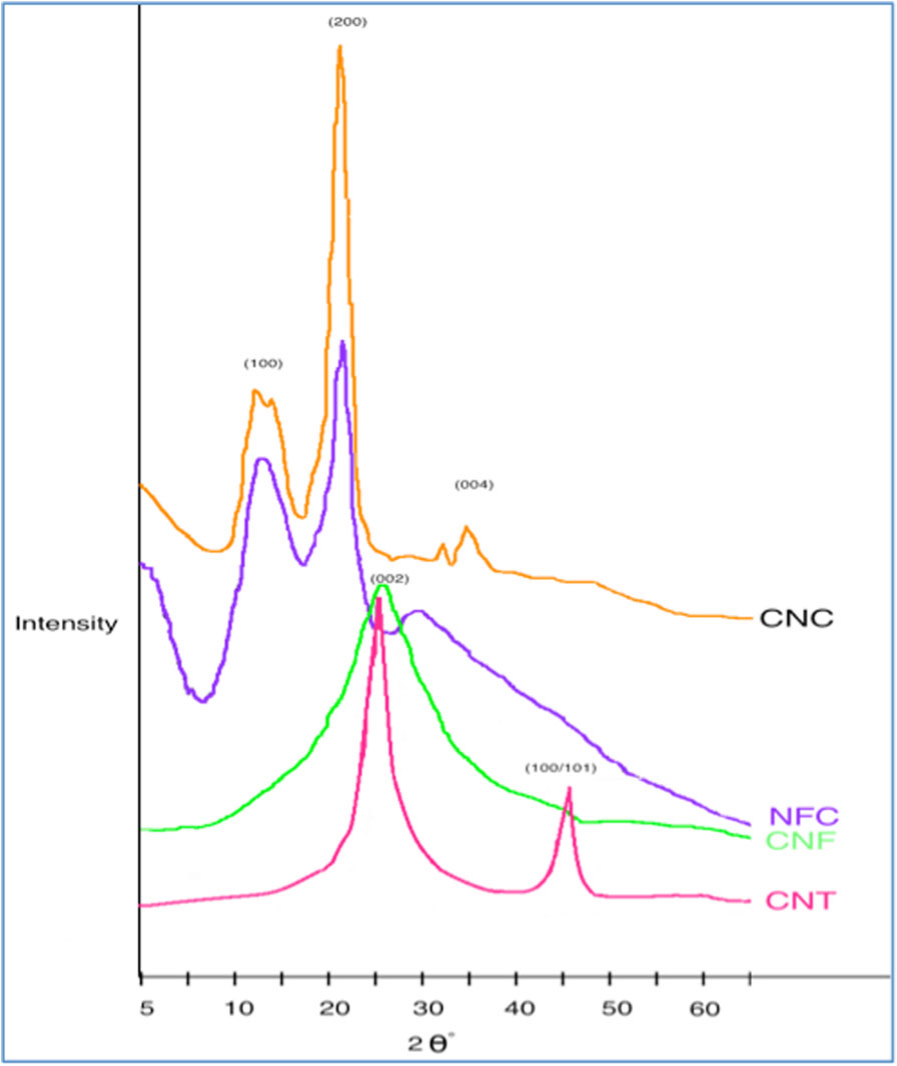
XRD pattern of nanomaterials

X-ray diffraction (XRD) presented in Fig. 2, is a technique for determination of the atomic arrangements within a crystal. Three well-defined crystalline peaks typical of cellulosic nanomaterials were present at around 2θ = 15°, 22.5°, and 35°. It can be seen that the peak for the CNC at 2θ = 22.5° was significantly sharper than the peak for NFC. This was due to the existence of a higher crystalline domain of CNC than NFC.

For the carbonic nanostructures, the strongest diffraction peak at the angle (2θ) of about 20–30° ranges can be indexed as the C(002) reflection of the hexagonal graphite structure. The sharpness of the peak at the angle (2θ) of 25.5° indicates that the graphite structure of the CNTs was without significant damage since any decrease in the order of crystallinity in CNFs will make the XRD peaks broader. The other characteristic diffraction peaks of graphite at 2θ of about 43° was associated with C(100) diffractions of graphite.

### Thermal Resistance

**Fig. 3:**
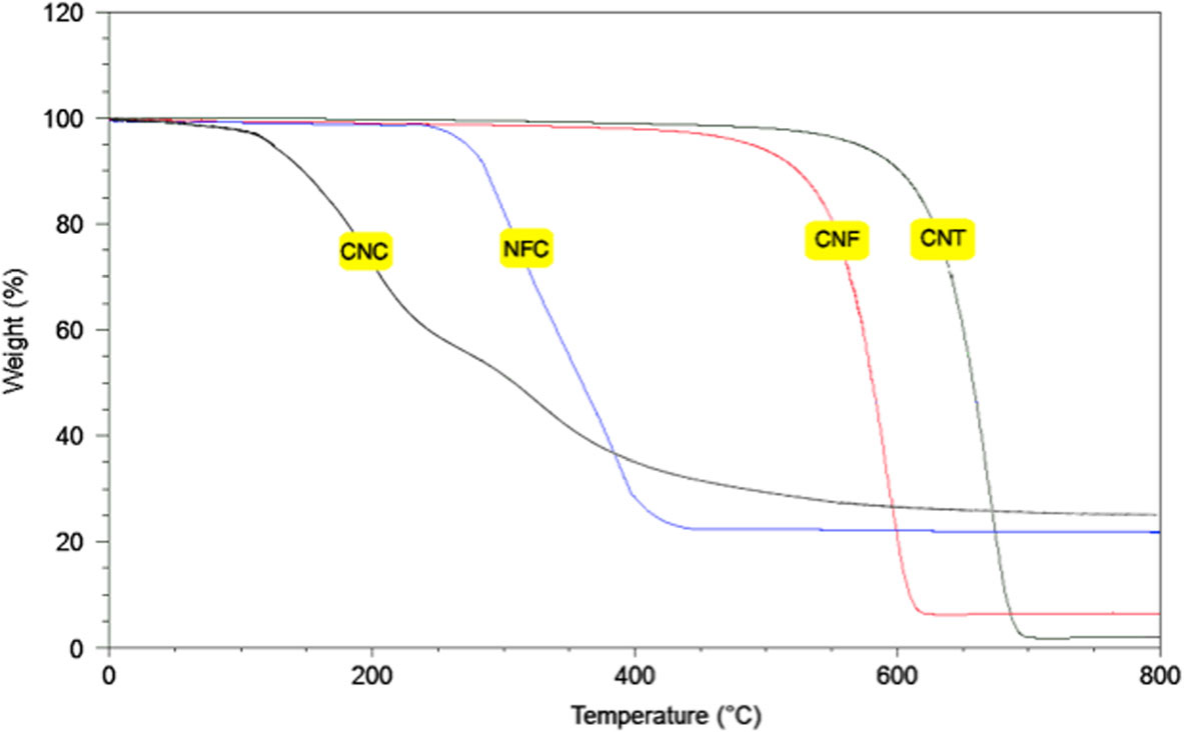
TGA curves of CNC, NFC, CNF, and CNT

In the TGA process, when the materials absorbed a certain amount of heat, a single degradation step for all samples and also thermal degradation began to occur. The degradation process led to the breakdown of the matrix structure of the sample. The TGA diagrams in Fig. 3 illustrate the nanomaterials degradation based on the weight loss (wt.%) versus temperature (°C). The temperature where the weight loss exhibited 5 wt.% was defined as the onset decomposition temperature (T_onset_) while the temperature at which the degradation rate reached a maximum was defined as Tmax. For CNC, two degradation processes were evident at around 180 °C and 300 °C while NFC showed only one pyrolysis process at 300 °C that is typical of cellulose. These indicated that the thermal stability of CNCs prepared by sulfuric acid hydrolysis was lower than that NFC produced by mechanical technique. The lower temperature process may correspond to the degradation of highly accessible and therefore more highly sulphated amorphous regions, whereas the higher temperature process was related to the breakdown of unsulphated crystals. The presence of acid sulphate groups decreased the thermal stability of cellulose as a result of the dehydration reaction [30]. Besides, CNC with the high surface area has a higher heat-transfer rate which leads to decrease the thermal stability.

On the other hand, it was obvious that the decomposition temperature of the CNF started about 480 °C and completed at 615 °C, whereas for CNT, the decomposition temperature of the sample was increased to about 600 °C and completed at 690 °C. Since the composition of CNT and CNF is similar, therefore, the higher thermal stability of CNT than CNF due to its size, structure, and morphology. Although the CNT has more surface area in comparison with CNF, CNT with stronger structure has higher thermal stability. Briefly, the TGA results revealed that the thermal degradation of the synthetic nanomaterials (CNF and CNT) are much lower than natural nanostructures (CNC and CNF). Therefore, synthetic nanomaterials, especially CNT with high thermal stability has the ability to be used in thermal devices.

### Electrochemical Results

**Fig. 4:**
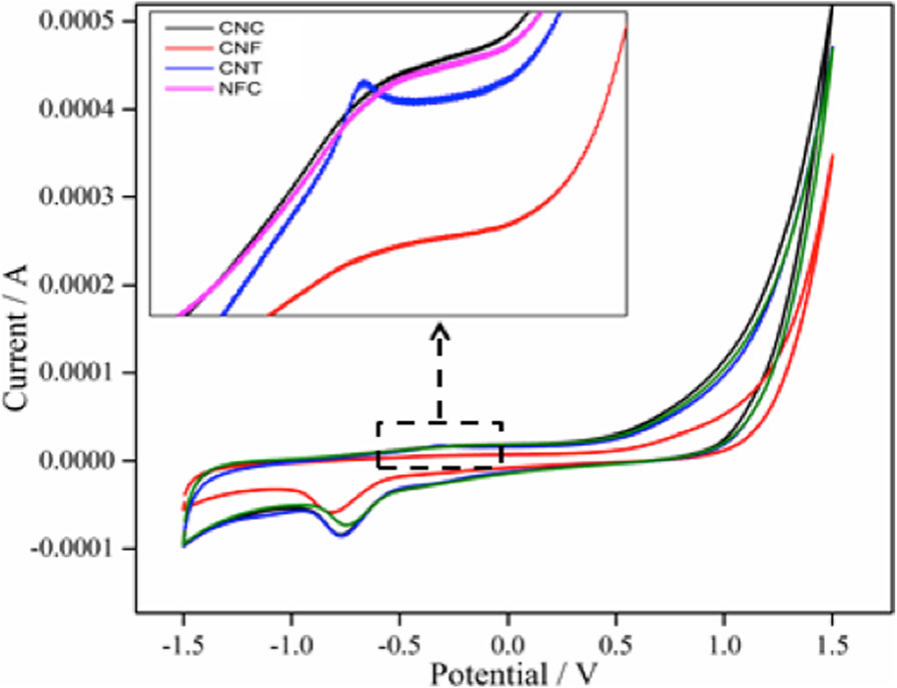
Cyclic voltammograms of CNC, CNF, CNT, and NFC in PBS buffer solution (pH 7.0). Scan rates: 0.1 Vs^−1^

The cyclic voltammograms (CV) of SPE, the natural and synthetic nanomaterials are presented in Fig. 4. The voltammograms of the nanomaterials showed that the rectangular peaks with redox peaks demonstrating the contribution of electrochemical double layer capacitor (EDLC) manner and pseudocapacitance impact.

The increases in cyclic areas of the synthetic nanomaterials in comparison with natural nanomaterials are related to the increase of storage capacitance of the CNF and CNT electrodes, and it could be possibly due to this fact that porosity and surface area of the synthetic nanomaterials are much more than natural nanomaterials. The redox peaks in synthetic nanomaterials showed that CNF and CNT could speed up the electrochemical reaction and supply an outstanding way for the charge transfer. Besides, the synthetic nanomaterials enhanced the output of electrode by creating a higher surface area and decreasing the current flow resistance. The presence of the plateau at about − 0.5 to 0.5 V can be attributed to the formation of a solid electrolyte interface (SEI) film on the surface of CNC and NFC electrodes.

### Cytotoxicity Analysis

MTT assay was used for testing cell viability of the nanomaterials. The relative cell viability (%) related to control wells containing cell culture medium without nanoparticles was calculated by the following equation:

$$ \frac{\left[A\right]\mathrm{test}}{\left[A\right]\mathrm{control}}\times 100 $$

**Fig. 5:**
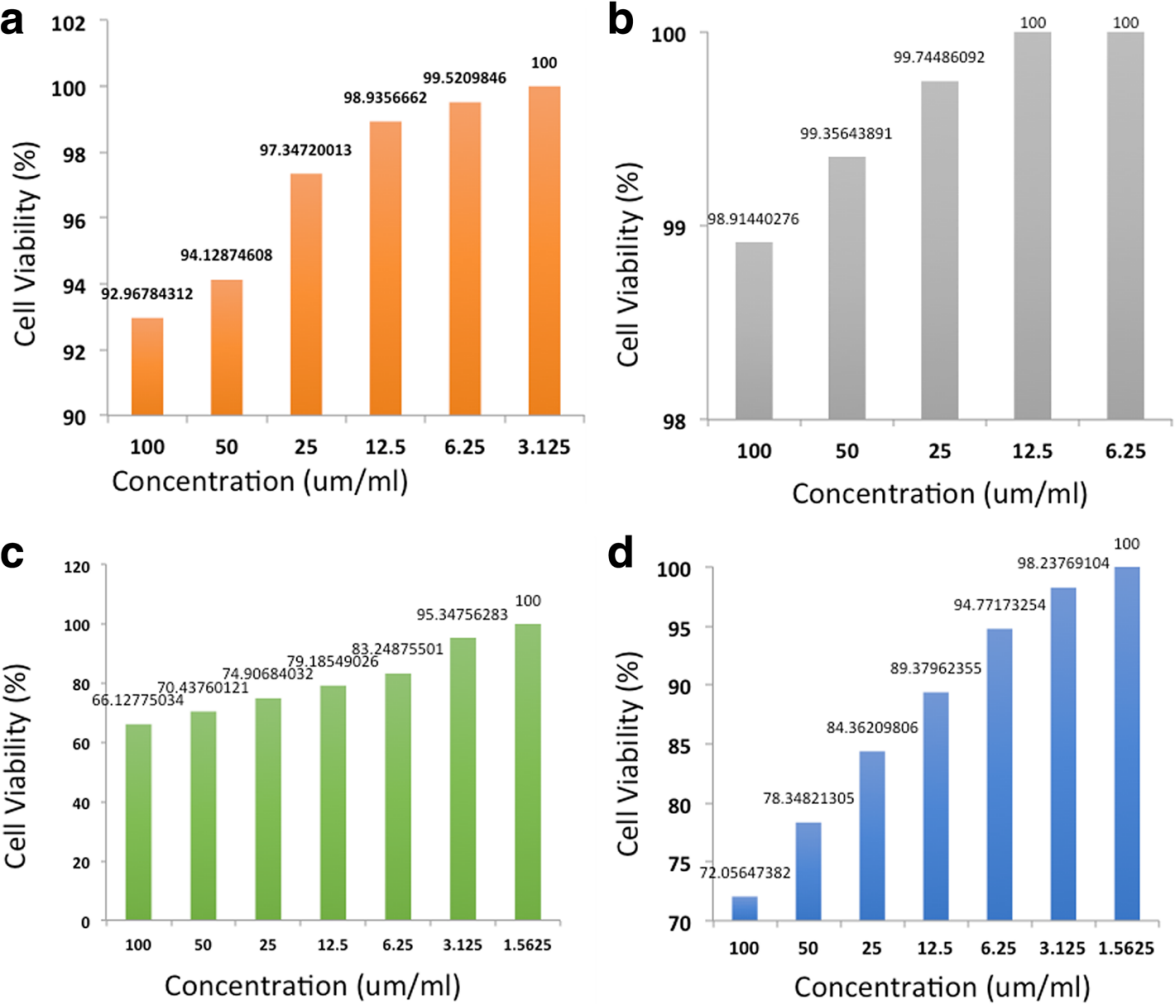
The cytotoxicity analysis of **a** CNC, **b** NFC, **c** CNF, and **d** CNT

Based on the results shown in Fig. 5, natural nanomaterial compound was found less toxic on 4T1 breast cancer cell line compared to the synthetic nanomaterials compound. The NFC and CNC compound inhibited/killed about 1.1 and 7% of cells at a concentration of 100 μg/ml whereas, at a similar concentration, the carbon nanofiber and nanotubes killed the cells at a higher percentage (34 and 28%, respectively). At the concentration of 12.5 μg/ml, NFC did not reveal any toxicity against the cell as the cells were 100% viable, whereas, it was not the case for CNC compound as it killed 7% of living cells. Besides, CNC had no toxicity effect at 3.125 μg/ml while, at this concentration CNF and CNT killed 4.3 and 1.7%, respectively. Therefore, natural nanomaterials are better choices for biomedical applications rather than synthetics nanostructures.

## Conclusions

In this research, natural nanomaterials (CNC and NFC) were produced by using acid hydrolysis and mechanical techniques and also synthetics nanostructures (CNF and CNT) via CVD method. SEM, TEM, and XRD methods not only confirmed the crystalline nature of CNC and high graphitization structure of CNT but also determined the smaller diameter against NFC and CNF. Besides, EDX proved the high purity of the nanomaterials. Moreover, BET surface area analyzer found that synthetic nanomaterials had much more surface area than natural nanomaterials.

The properties of the produced nanomaterials such as electrochemical properties, thermal resistance, and cytotoxicity effects on living cells were investigated and compared, comprehensively. Hence, the influences of nanomaterial morphology on their properties were studied. Regarding the obtained results, the synthetic nanoparticles had higher thermal resistance and storage capacity compared to natural nanomaterials while natural nanomaterials with lower cytotoxicity effects on living cells had more potential to be used in biomedical applications.

## Methods

Materials and Methods should be described with sufficient details to allow others to replicate and build on published results. Please note that publication of your manuscript implicates that you must make all materials, data, computer code, and protocols associated with the publication available to readers. Please disclose at the submission stage any restrictions on the availability of materials or information. New methods and protocols should be described in detail while well-established methods can be briefly described and appropriately cited.

### Preparation of Natural Nanofibers

Cellulose was isolated from kenaf bast fiber by the method adapted from Kargarzadeh et al. (2012) [30]. Here, CNC and NFC were produced from cellulose kenaf bast fiber by using acid hydrolysis and mechanical methods, respectively. The CNC was isolated through the method reported by Kargarzadeh and co-authors in 2012 using acid hydrolysis conducted at 65% of aqueous H_2_SO_4_ under mechanical stirring at 50 °C for 40 min [30]. Then, the suspension was cooled and diluted with distilled water (10 °C) and then centrifuged with 10,000 rpm for 10 min for three times. After that, it was dialyzed with distilled water until reaching a fixed pH. In order to disperse the nanocrystals, ultrasonic treatment was carried out. After adding several drops of chloroform to prevent bacterial growth, the resulting suspension was subsequently stored in the refrigerator.

For fabricating NFC, water retted kenaf bast fibers coded as RF were cut into short pieces and then cooked in a JSR-212 rotatory digester with 25 wt% NaOH and 0.1 wt% anthraquinone solution (liquor to fiber ratio was 7:1) at 160 °C for 2 h. Anthraquinone was added to the cooking liquor to enhance the delignification rate and also protect the fibers from alkali degradation and so-called end-wise degradation of cellulosic chains.

### Preparation of Synthetics Nanofibers

In this part, the nickel nitrate hexahydrate powder (Ni(NO_3_)_2_.6H_2_O) was put as precursors for Ni catalyst in a quartz boat located in the CVD reactor and then dried at 160 °C to remove humidity for 50 min and then increased the temperature to 400 °C to remove nitrate compounds for 1 h. In this step, the resultant Ni particles as catalysts were produced. In order to synthesis the synthetics nanofibers, the CVD reaction temperature must be altered [10, 31]. The temperature was fixed at 650 °C and 800 °C to fabricate CNF and CNT with high quality, respectively. The process was conducted by the decomposition of the acetylene at 50 sccm flow rate on the Ni particles at 100/100 sccm H_2_/N_2_ flow rates for 30 min. In order to omit the catalyst from the fabricated carbon nanomaterials, a mixture of FeCl3 (1 M)/HCl (1 M) was used and carbon nanomaterials were poured into it followed by filtering. Then it was washed through distilled water several times and finally dried.

### Characterization of Synthesized Nanomaterials

#### Microscopy

Scanning electron microscopy (SEM), energy dispersive X-ray spectrometer (EDX), and transmission electron microscopy (TEM) were used to analysis the morphology, structure, and composition of nanomaterials, respectively.

#### X-ray Diffraction Analysis (XRD)

A nondestructive characterization technique that reveals some required information on the interlayer spacing, the structural strain, and the impurities of the product is the X-ray diffraction (XRD) analysis. For nanocellulose, XRD analysis indicates a peak of C (002) with different intensity indexed (CrI). The amorphous part was measured as the lowest intensity at a diffraction angle of around 2θ = 18.0°. On the other hand, carbon nanostructure exhibits a XRD pattern consisting of a few broad bands located near (002; 2θ = 25) and (100; 2θ = 45) reflections of hexagonal graphite structure and diffractions of graphite, respectively.

#### BET Surface Area Analysis

According to ISO 9277, the Brunauer, Emmett, and Teller (BET) method was used to calculate the specific surface areas of the nanomaterials using an adsorption instrument (BELSORP-mini II analyzer).

#### Electrochemical Analysis

PGSTAT204 system was employed to fulfill the electrochemical analysis. Besides, cyclic voltammetry was applied to evaluate the electrochemical behavior of modified electrodes with nanomaterials on the screen-printed electrode (SPE) in a buffer solution with 100 mVs^−1^ scan rate. Initially, the homogeneous suspension (2 ml deionized water/1 mg nanomaterial powder) was sonicated for 6 min. Then, a drop casting of 10 μl suspension onto the SPE made modified electrodes. The current-voltage (CV) diagrams of the samples were evaluated in − 1.5 to 1.5 V potential at room condition.

#### Cytotoxicity Analysis

In order to analysis the cytotoxicity potentials of the different nanomaterials and cell viability, the 3-[4,5-dimethylthiazol-2-yl]-2,5 diphenyltetrazolium bromide (MTT) dye reduction was used. The cytotoxic effect of the nanomaterials could be measured by using this assay based on the IC50 generated. A 100 μL of 4T1 cells at 0.8 × 105 cells/well concentration was poured into a 96-well plate and was kept in the RPMI medium for 24 h. The next day, natural and synthetic nanomaterials were added to the wells and then incubated for 72 h. MTT solution (5 mg/ml) (Calbiochem) was added, separately, at a volume of 20 μL into each well and was incubated for 3 h. Later, the solutions were removed from wells and 100 μL of DMSO was added to solubilize the formazan crystals. Finally, the plate was read using an ELISA plate reader at a wavelength of 570 nm (Bio-Tek Instruments, USA).

#### Thermogravimetric Analysis (TGA)

To analyze the thermal resistance, a thermogravimetric analysis (TGA) was used. TGA was done by a Mettle Stare SW 9.10 thermal gravimetric analyzer. Initially, 0.5 mg of the nanomaterials was located in the crucible located into the system of TGA and heated to about 200 °C for 5 min to eliminate humidity. After that, the heating program was increased to 600 °C and 900 °C with the rate of 10 °C/min at the presence of N_2_ flow for natural nanofibers and synthetics nanofibers, respectively.

## Abbreviations

BET: Brunauer, Emmet, and Teller
CNC: Cellulose nanocrystal
CNF: Carbon nanofiber
CNT: Carbon nanotube
CV: Cyclic voltammograms
CVD: Chemical vapor deposition
EDLC: Electrochemical double layer capacitor
EDX: Electron dispersive X-ray
NFC: Nanofiber cellulose
SEI: Solid electrolyte interface
SEM: Scanning electron microscopy
TEM: Transmission electron microscopy
TGA: Thermogravimetric analysis
XRD: X-ray diffraction
